# Optical Characterization of Neurosurgical Operating Microscopes: Quantitative Fluorescence and Assessment of PpIX Photobleaching

**DOI:** 10.1038/s41598-018-30247-6

**Published:** 2018-08-22

**Authors:** Evgenii Belykh, Eric J. Miller, Arpan A. Patel, Baran Bozkurt, Kaan Yağmurlu, Timothy R. Robinson, Peter Nakaji, Robert F. Spetzler, Michael T. Lawton, Leonard Y. Nelson, Eric J. Seibel, Mark C. Preul

**Affiliations:** 1 Department of Neurosurgery, Barrow Neurological Institute, St. Joseph’s Hospital and Medical Center, Phoenix, Arizona USA; 2 University of Washington, Human Photonics Lab, Seattle, Washington USA; 3 RGB Optics, Lake Forest Park, WA USA

## Abstract

Protoporphyrin IX (PpIX) induced by 5-aminolevulinic acid (5-ALA) is increasingly used as a fluorescent marker for fluorescence-guided resection of malignant gliomas. Understanding how the properties of the excitation light source and PpIX fluorescence interact with the surgical microscope is critical for effective use of the fluorescence-guided tumor resection technique. In this study, we performed a detailed assessment of the intensity of the emitted blue light and white light and the light beam profile of clinical grade operating microscopes used for PpIX visualization. These measurements revealed both recognized fluorescence photobleaching limitations and unrecognized limitations that may alter quantitative observations of PpIX fluorescence obtained with the operating microscope with potential impact on research and clinical uses. We also evaluated the optical properties of a photostable fluorescent standard with an excitation-emission profile similar to PpIX. In addition, we measured the time-dependent dynamics of 5-ALA-induced PpIX fluorescence in an animal glioma model. Finally, we developed a ratiometric method for quantification of the PpIX fluorescence that uses the photostable fluorescent standard to normalize PpIX fluorescence intensity. This method increases accuracy and allows reproducible and direct comparability of the measurements from multiple samples.

## Introduction

Operating microscopes are commonplace within the neurosurgical operating theater and are a mainstay of surgical procedures for brain tumor removal^1^. As the most important visualization tool in daily use for neurosurgery, operating microscopes are gaining advanced functionality by means of innovative illumination modes. To ensure surgical success, the neurosurgeon must fully understand the illumination properties and functionality of the microscope, especially within the context of fluorescence-guided tumor resection.

The principle of fluorescence-guided tumor resection relies on the use of targeting agents with fluorescent properties that can be administered to patients before or during surgery. These agents are intended to accumulate within and around the tumor tissue or within the cells of the tumor, depending on the selectivity and actions of the fluorophore. The desired diagnostic result is to augment visual differentiation and detection of the tumor tissue margins during surgery based on fluorescence. The most notable recent example of a fluorescent agent developed for tumor detection in neurosurgery is 5-aminolevulinic acid (5-ALA), which is used to indicate the presence of tumors and the border regions of malignant gliomas^2^.

5-ALA was recently designated as the first US Food and Drug Administration–approved agent for fluorescence-guided resection of high-grade gliomas^3^. This prodrug results in fluorescent PpIX accumulation in tumors through a distortion in the metabolic conversion of 5-ALA to heme^4^. Understanding both the nuances of its fluorescent properties and the effects that occur with changes in excitation intensity and duration of light exposure is critical to optimizing the intraoperative utility of PpIX as a guide for the surgeon to discriminate the border region of the tumor. The process of photobleaching and the reduction of PpIX fluorescence that occurs are believed to be directly related to the light intensity of the operating microscope and the duration of exposure^5,6^. However, the rate at which the fluorescence intensity of PpIX declines with exposure to light has not been thoroughly investigated for commercial grade operating microscopes for neurosurgery.

Commercial grade operating microscopes are increasingly outfitted with modules for fluorescence emission detection at various wavelengths. These special illumination modules have become commonplace during neurosurgery for cerebrovascular disorders and are increasingly used for brain tumor resection procedures. Comprehension of the microscope illumination output^7^, fluorescence, and photobleaching can have a profound influence on the suitable protocol a neurosurgeon will follow for tissue resection. The extent of resection for both low-grade and high-grade gliomas has a weighty impact on patient life expectancy^8–10^. These principles should be understood not only by physicists and manufacturers but also by neurosurgeons, who need to be informed about the capabilities and limitations of the fluorescent microscope modules that may be used in surgery and in other treatment procedures for brain tumors.

The development of standardized methods thus becomes increasingly important for clinical trials and studies that obtain measurements or observations using the surgical microscope in intraoperative fluorescent modalities during the resection of neoplastic brain tissue. Previous studies have mostly used qualitative fluorescence intensity grading, as it appears in the oculars of the microscope, to compare with the histological grading of the tissue^11–14^. Recent studies have advocated for the detailed quantitative analysis of fluorescence for improved accuracy of fluorescence guidance and identification of tumor tissue^15–19^. However, conventional operating microscopes were not originally designed for quantitative fluorescence measurement, and multiple parameters that can influence fluorescence detection and measurement have not been well described.

Because of the significance of the 5-ALA FDA approval combined with clinical grade microscope fluorescence modules, we performed detailed measurements of the emitted light intensity and illumination beam profile for several commercial neurosurgical operating microscopes used for PpIX visualization. As we describe herein, fluorescence-guided surgery does not rely on a simple method, as there are multiple aspects of the inter-relationship of biology and imaging technology to consider. In investigating principles of standardization for fluorescence guidance, we developed and assessed the optical properties of a photostable fluorescent standard with an excitation-emission profile similar to PpIX. In addition, we measured the time-dependent dynamics of 5-ALA–induced PpIX fluorescence in an animal glioma model. Finally, we developed a method for quantification of PpIX fluorescence that uses photostable fluorescence standards and allows reproducible results and direct comparability of the measurements from multiple samples.

## Results

### Operating Microscope Optical Power Measurements

**Figure 1:**
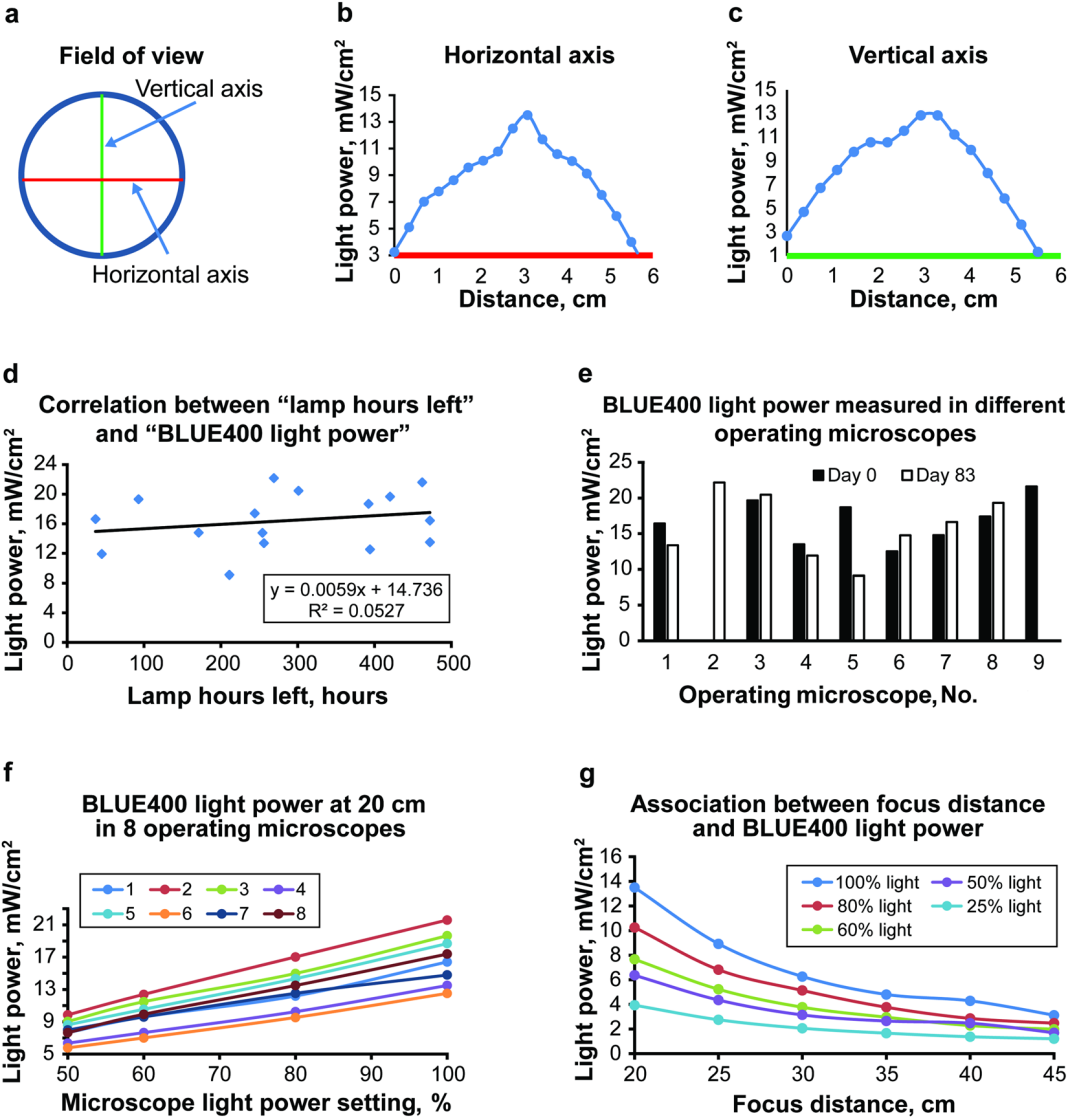
Optical power measurement experiments. Incident BLUE 400 optical power profile across the field of view of the operating microscope. Unless otherwise specified, measurements were performed at 20-mm focus distance and 100% microscope light power settings. (**a**) Diagram showing where the measurements were taken. (**b**) Light intensity profile across the horizontal axis of the field of view. (**c**) Light intensity profile across the vertical axis of the field of view. (**d**–**f**) Results of optical power assessment with different operating microscopes. (**d**) Graph showing absence of correlation between the remaining lamp hours and optical power measured at the center of the field of view. (**e**) Graph showing results of repetitive optical power measurements in operating microscopes spanning 83 days. There was no clear association with use time. Pentero microscopes 2 and 9 were assessed only once because of their limited availability. (**f**) Graph showing association between the microscope optical power setting and the measured incident optical power at a 20-cm focus distance in 8 operating microscopes. (**g**) Graph showing association between the focus distance and the measured incident optical power at 5 different microscope light power settings. *Used with permission from Barrow Neurological Institute*, *Phoenix*, *Arizona*.

The white light and blue light (BLUE 400 fluorescence mode) optical power profile of all the operating microscopes that we evaluated varied across the field of view (FOV). The distribution of light across the horizontal and vertical axis was bell-shaped (Fig. 1a–c). The profile of light intensity was slightly skewed along the vertical axis toward one side because of the angulation of the microscope relative to the surface.

Interestingly, further analysis revealed no correlation between the “lamp hours left” value and the blue light optical power (Fig. 1d,e). As the “lamp hours left” parameter changed, the degree of change in the intensity of the blue light optical power became inconsistent.

We also analyzed the effect of focal distance and the light power settings on the measured light power density across the FOV. Analysis revealed that the measured blue light optical power density at a focus distance of 20 cm had a direct and positive correlation with the light power settings of the microscope (Fig. 1f). A similar correlation was found at a focus distance of 30 cm (Supplemental Fig. 1). Further analysis revealed, as expected, an inverse relationship between focus distance and measured light power density in the BLUE 400 operating mode (Fig. 1g). Optical power measurements using the white light mode showed a similar association between the focus distance and the optical power density among various light power settings (Supplemental Fig. 2). For example, when the microscope is operated in BLUE 400 mode at 100% light power and with a 30-cm focus, the optical power density is roughly equivalent to operating the same microscope in BLUE 400 mode at 50% light power with a 20-cm focus distance. The relationship between focus distance and measured light power density was non-linear for both blue and white light illumination.

### Fluorescence Measurements of Dye-in-Polymer standards

**Figure 2:**
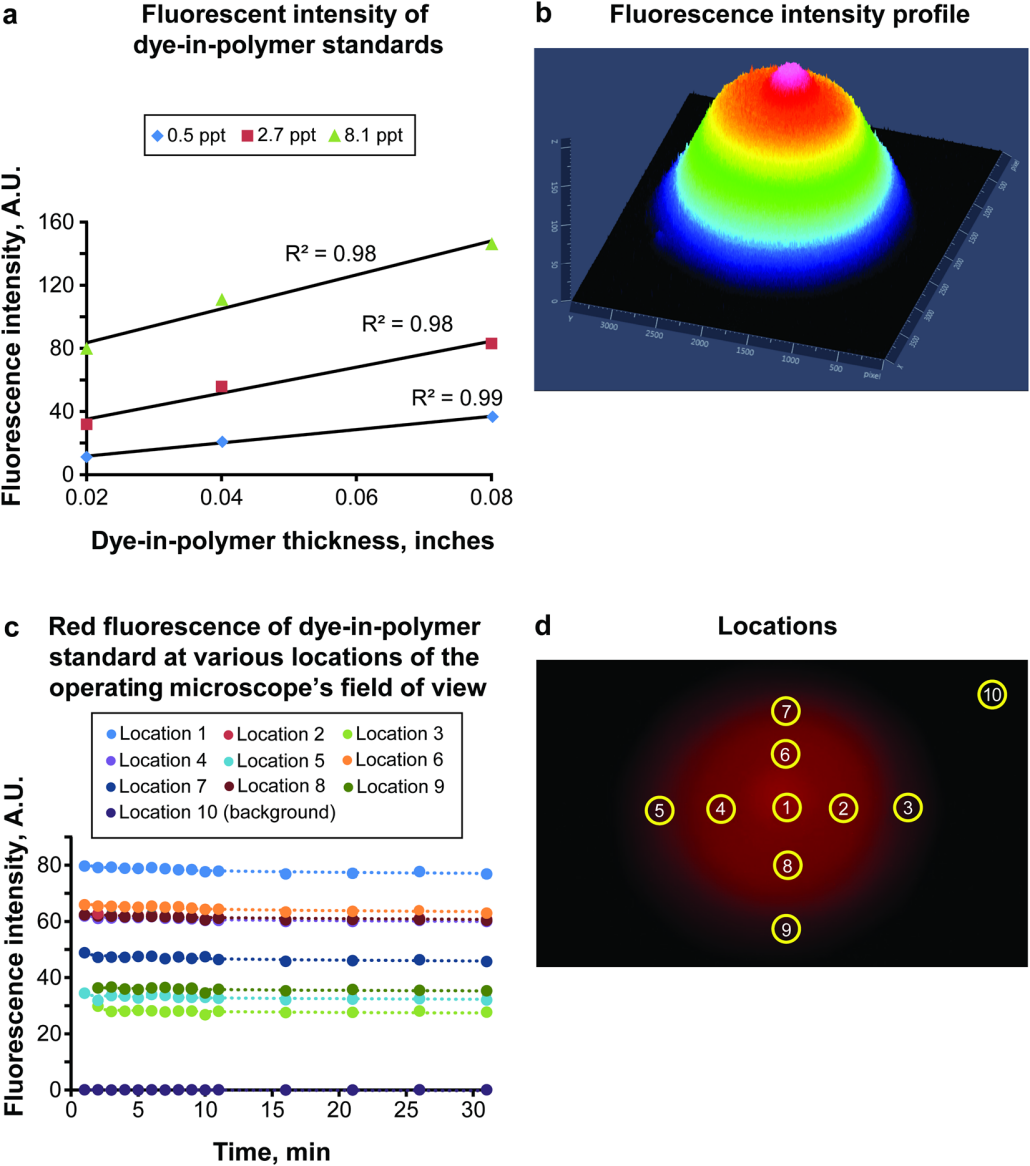
Fluorescence in dye-in-polymer standards. (**a**) Fluorescent intensity in each of the 9 standards at time zero, labeled by dye concentration and thickness of the sheet. The intensity value increased in a linear fashion as thickness was increased for each specific concentration. This rate of linear increase varied depending on the concentration of the fluorescent dye in a standard. (**b**) Three-dimensional diagram of the fluorescence intensity of standard 7 under the beam of BLUE 400 light. The intensity is plotted in Z axis and color coded, the X and Y axes are on the surface. (**c**) Rate of photobleaching of 10 regions over the course of 30 min for standard 7. (**d**) Image of the dye-in polymer standard sheet under the operating microscope BLUE 400 light showing 10 uniform size regions (1–10) that were selected for intensity analysis (shown in **c**). *Ppt*, *parts-per-thousand by weight*; *A*.*U*., *arbitrary units*. *Used with permission from Barrow Neurological Institute*, *Phoenix*, *Arizona*.

The starting fluorescence intensity for each standard varied by the concentration of the dye and polymer thickness. The intensity value increased in a linear fashion with increased thickness for a specific concentration. However, the rate of linear increase also varied by the concentration of the dye. For instance, the rate that the intensity gained as thickness increased for the 8.1 parts-per-thousand (ppt) by weight standards was greater than the rate of increase in the 0.5 ppt standards (Fig. 2a).

### Fluorescence Across the Field of View is Non-Uniform

**Figure 3:**
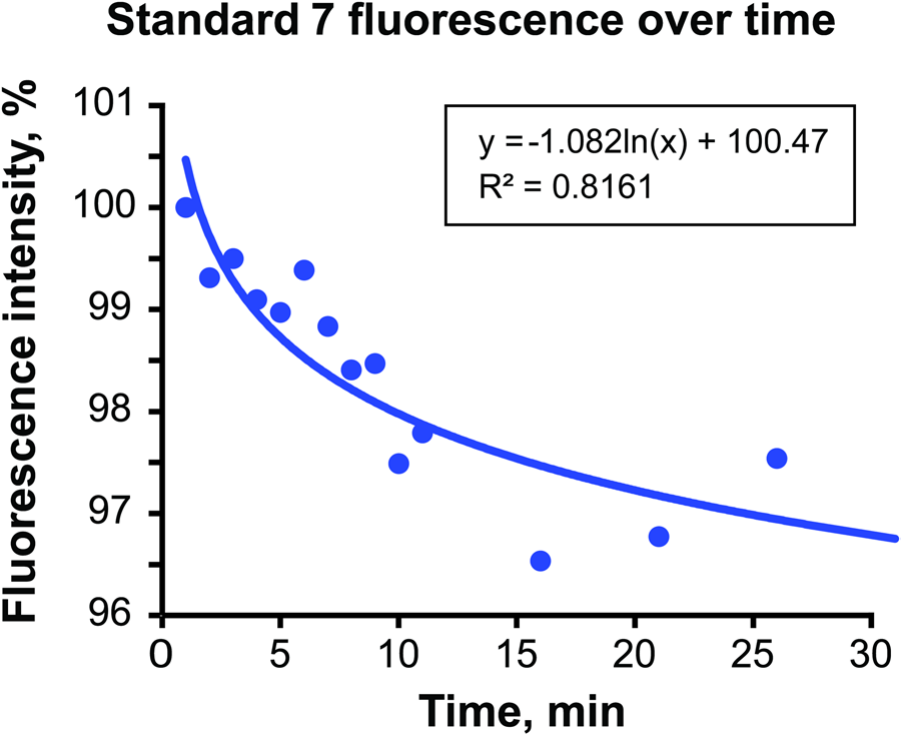
Fluorescence decay in dye-in-polymer standards. Logarithmic fluorescence decay at location 1 on standard 7 over 30 min. Logarithmic regression equation with R^2^ value is included. *Used with permission from Barrow Neurological Institute*, *Phoenix*, *Arizona*.

Quantitative analysis of the acquired images revealed that a lack of uniformity in the fluorescence intensity across the FOV of any of the standard materials (Fig. 2b). The pattern of fluorescence intensity correlated with the intensity of the illumination blue light to which the standard was exposed at a given location. The non-uniform pattern was also partly due to the tilted position of the microscope. In addition to the non-uniform distribution of baseline fluorescent intensity across the FOV, the slow rate of photobleaching varied with light intensity. Images acquired over the course of 30 min were analyzed at 10 manually selected regions of interest (ROI), resulting in different rates of photobleaching (Fig. 2c,d). The coefficients of the natural logarithmic equations used to model the decay rate of each location for standard 7 and the Cox and Snell pseudo-R^2^ values are presented in Table 1. The data reveal that the central location (ROI 1, Fig. 2d), which was found to have the highest baseline fluorescent intensity, also has the greatest rate of photobleaching. The area on the periphery (ROI 10) showed the lowest intensity at the beginning and the lowest rate of photobleaching. Although the fluorescence decay was different among different ROIs in the FOV, such fluorescence decay followed the logarithmic pattern. Overall, the photobleaching rate was similar across standards 1–9 and was extremely low (Fig. 3). However, not every data set for each standard analyzed generated a logarithmic equation with high pseudo-R^2^ value due to a multitude of reasons, including outliers and minuscule changes of fluorescence intensities with time. The total amount of photobleaching that occurred over the 30-minute exposure for any given standard was consistently a small percentage of the total fluorescent intensity. The small total amount of decay over the span of 30 min leaves the intensity calculations vulnerable to natural variations in camera imaging exposure times, as well as fluctuations in the total amount of light present within the imaging room.

**Table 1:** Logarithmic models of photobleaching decay in dye-in-polymer standard*.

Location	Coefficient of Natural Logarithm Equation (y = c*ln(x) + y_0_)	R^2^ Value
1	−0.862	0.81609
2	−0.829	0.79666
3	−0.608	0.73443
4	−0.59	0.83507
5	−0.579	0.73426
6	−0.736	0.73479
7	−0.438	0.384
8	−0.461	0.34509
9	−0.469	0.34686
10	−0.0002	<0.001

*Dye-in-polymer standard 7 was used for this table. Locations 1–10 are represented in Fig. 2. Location 1 is the center with maximum intensity and 10 is the background, outside the circle of fluorescence.

Overall, our results confirm that the logarithmic nature of photobleaching kinetics in the polymer standards is similar to that observed in the tumor PpIX. However, in contrast to PpIX, this decay was minimal for the standards.

### Comparison of Internal Microscope Camera and External Camera with Red Filter

**Figure 4:**
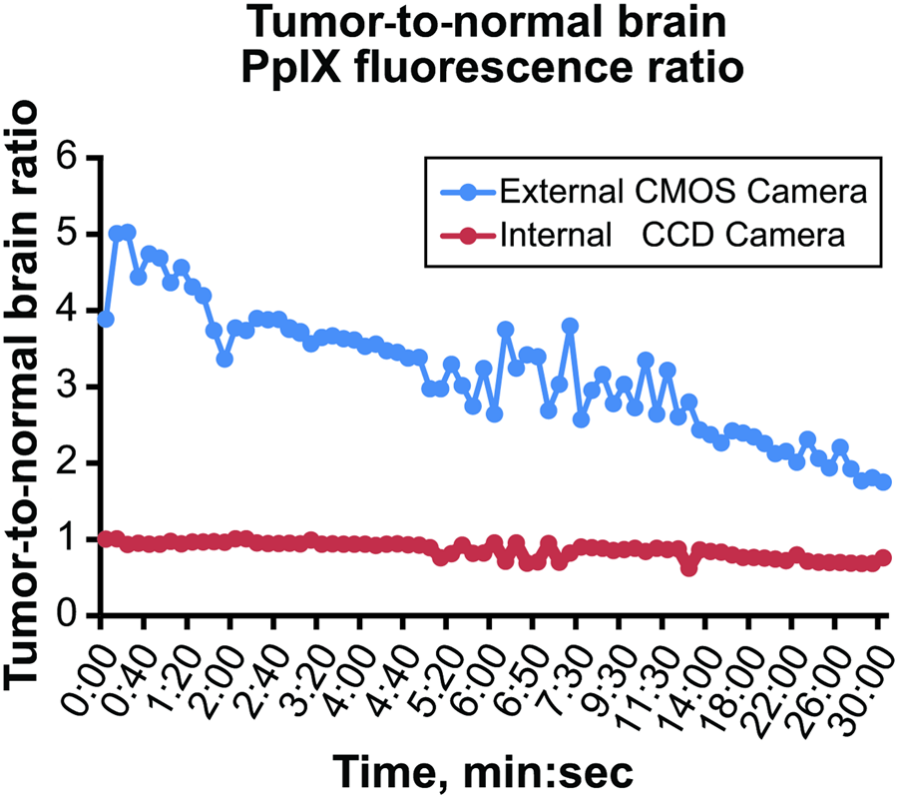
Tumor-to-background ratios calculated from averages of all tumor and background fluorescence values captured using the external CMOS camera or the internal CCD camera of the operating microscope. The internal camera was not sensitive enough to record meaningful fluorescence data. The averages were calculated from 6 different samples for each time point. *PpIX*, *protoporphyrin IX; CMOS*, complementary metal–oxide–*semiconductor; CCD*, *charge-coupled device*. *Used with permission from Barrow Neurological Institute*, *Phoenix*, *Arizona*.

Images taken with the external Canon camera showed a declining tumor-to-background ratio over the 30 min of observation in the murine gliomas. This was not observed with images taken using the internal operating microscope camera. In fact, these images showed background brain tissue fluorescence as brightly as it showed the tumor tissue (Fig. 4).

The tumor-to-background ratios calculated from the external camera were significantly higher than those calculated with an internal microscope camera (p < 0.001). Typical color charge-coupled device (CCD) cameras do not have adequate out-of-band rejection filters, which allows blue light to spill over into the red channel and compromise the ability of the camera to measure the tumor-to-background ratio. However, the additional red glass filter mounted in front of the Canon camera lens offers more than 1000× optical density attenuation of blue reflected light. Hence, the red channel tumor-to-background measurements recorded by the Canon camera system are not corrupted by blue light.

### Subjective Grading of Fluorescence Intensity of Dye-in-Polymer Standards

Subjective grading of the fluorescence intensity of the standards by four observers exhibited a moderate degree of intrarater agreement (69 ± 14%) between the first and second grading attempts (Table 2). A second grading attempt was used to compare interobserver agreement. The interobserver agreement was 81.5%, fixed marginal kappa was 75.1%, and free marginal kappa was 76.9% (Table 3). Standards number 1, 4, 6, and 9 were selected for further experiments with biological tissue as they covered the full dynamic range of PpIX fluorescence intensity and exhibited good interobserver agreement.

**Table 2:** Intrarater agreement for the fluorescence intensity score of dye-in-polymer standards.

Observer	Percentage of Agreement Between First and Second Grading Attempts
NS 1	67%
NS 2	56%
MS 1	67%
MS 2	89%
Mean ± SD	69 ± 14%

NS = neurosurgeon; MS = medical student.

**Table 3:** Interrater agreement for the fluorescence intensity score of dye-in-polymer standards.

Dye-in-polymer Standard, No.	Observer’s Score*				% Agreement
	NS1	NS2	MS1	MS2	
1	4	4	4	4	100%
2	4	4	4	4	100%
3	3	4	3	4	50%
4	3	4	3	3	75%
5	3	3	3	3	100%
6	2	2	2	2	100%
7	2	2	2	2	100%
8	1	1	2	1	75%
9	1	1	1	1	100%

NS = neurosurgeon; MS = medical student.

*Score is based on Table 5 of methods section.

### Decay of PpIX Fluorescence in an Experimental Brain Tumor

**Figure 5:**
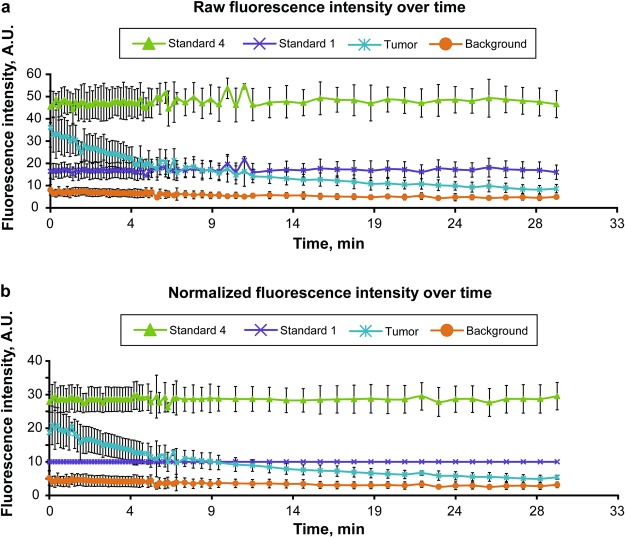
Dynamics of PpIX photobleaching in the tumor and dye-in-polymer standards. (**a**) Raw data. (**b**) Data were normalized, using standard 1 as a 10% reference point. Presented as mean ± SD. *A*.*U*., *arbitrary units*. *Used with permission from Barrow Neurological Institute*, *Phoenix*, *Arizona*.

We have used the dye-in-polymer standards to normalize the PpIX fluorescence intensity from the tumors (Fig. 5). Fluorescence of standard 1 was considered as a nominal constant 10% fluorescence intensity. The tumor, background, and other standards were converted to percentage values relative to this 10% value for each time point (Fig. 5b). Decay of PpIX fluorescence in the tumor under illumination by BLUE 400 light occurred rapidly and could be modeled according to the logarithmic equation: y = −0.048 ln(x) +0.2877, R^2^ = 0.83. According to this rate of decay, the half-life was 14 min 25 sec when calculated using data points at time zero and 30 min.

**Figure 6:**
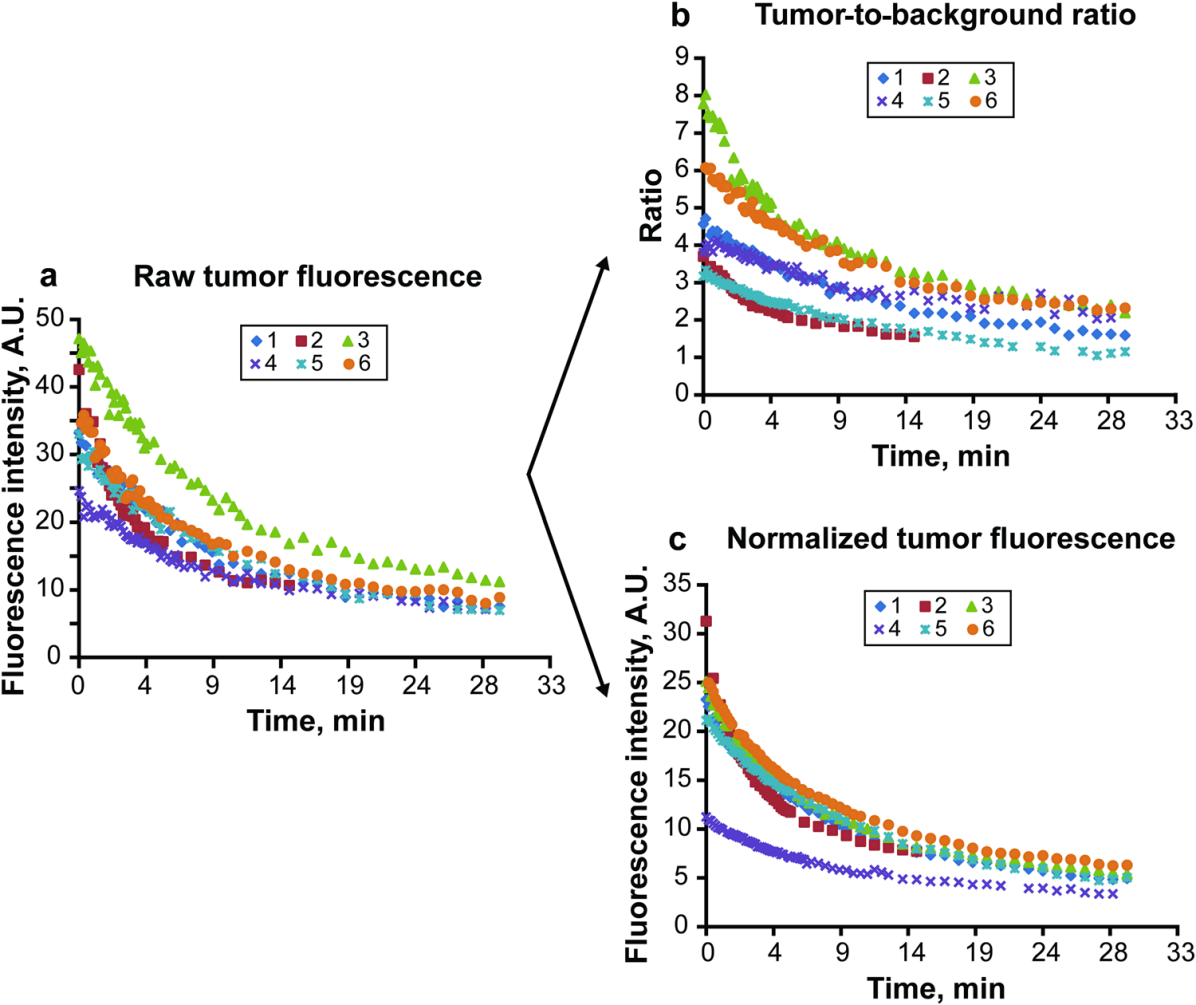
Quantitative assessment of PpIX fluorescence in six brain tumors under the operating microscope. Observation within 30 min of continuous BLUE 400 light. (**a**) Raw tumor fluorescence values. (**b**) Tumor-to-background ratios calculated from the raw data. (**c**) Calculated tumor fluorescence values after normalization to the standard 6. Normalization, which allowed for direct comparison of the PpIX intensities among specimens, revealed that tumor 4 had significantly lower absolute tumor fluorescence. *A*.*U*., *arbitrary units*. *Used with permission from Barrow Neurological Institute*, *Phoenix*, *Arizona*.

The tumor-to-background ratio decreased significantly from a mean (minimum to maximum) of 4.6 (3.2–7.8) at the beginning of the experiment to 2.8 (1.8–3.9) at the 10-min mark (overall 42% decrease, p < 0.01) in all specimens. Normal brain tissue also showed significant autofluorescence decay. The normal brain-to-background ratio of 5.2 ± 2.1 at time zero decreased to 3.7 ± 1.3 in 10 min (p = 0.02) and to 2.85 ± 1.00, at 30 min (p < 0.01). This background brain autofluorescence decay rate was 28% in 10 min, which was significantly faster than exhibited by any of the standards. All standards showed insignificant change in fluorescence (−0.3% in 10 min, p = 0.90; + 3.5% in 30 min, p = 0.99 for standard 1). The method of normalization using simultaneous recording of the fluorescence of the dye in the polymer standards significantly reduces biases when PpIX tumor fluorescence intensity is compared among different samples (Fig. 6).

## Discussion

### Importance of Quantitative Fluorescence Measurement

Although high-grade gliomas usually emit high levels of fluorescence, low-grade gliomas show a relatively lower amount of fluorescence after 5-ALA administration^20^. In addition, unlike in high-grade gliomas, PpIX fluorescence is reported to be patchy or lacking in some metastases^21^ and central nervous system lymphomas^22^ and rarely in meningiomas^23–25^ as visualized with the blue light of current operating microscopes. Visual PpIX fluorescence in gliomas is not uniform and is typically patchy with areas of high and low fluorescence intensity. This patchy appearance may be associated with inhomogeneous tumor growth, glioma cell diversity, overall tissue component heterogeneity (e.g., cysts and areas of necrosis), and differences in metabolism. The uneven nature of fluorescence distribution decreases precision in distinguishing tumor and normal tissues, especially at the critical border region. It has been shown that real-time quantification of the tumor fluorescence in human gliomas^15^ and more sensitive detection methods used in animal gliomas^26^ could facilitate tumor margin identification with more accuracy. Furthermore, since normal tissue exhibits overlapping red autofluorescence^6^, a method to precisely separate tumor and background autofluorescence can improve quantification of PpIX fluorescence^27^. The Canon camera red channel recordings could be used to subtract adjacent autofluorescence from PpIX emission in the tumor regions. However, our study demonstrated that the use of surrounding normal brain autofluorescence values as a background is suboptimal because of differences in fluorescence intensity across and within specimens and also because of photobleaching of the background signal over 30 min from 8.0 ± 2.3 to 4.4 ± 0.8 arbitrary units (decay to 36% at 14.4 J/cm^2^ or 9.5 min, Fig. 5). The current literature suggests that advanced methods for quantitative assessment of PpIX are clinically relevant^15,28–32^, therefore, the results of this study provide pertinent insights into the technical aspects of using commercial-grade neurosurgical microscopes for quantification of fluorescence.

### Method for Quantitative PpIX Fluorescence Assessment using Dye-in-Polymer Standards

Quantification of tumor PpIX fluorescence requires reliable detector systems. Our study demonstrated that the external Canon complementary metal-oxide semiconductor (CMOS) camera with a dedicated long-wave-pass (LWP) red filter outperformed the internal operating microscope CCD camera for this purpose. Indeed, CMOS and electron-multiplying metal oxide semiconductor (EMOS) cameras have better detection sensitivity and lower read-out noise than CCD systems^16,17^. However, even systems with enhanced detection sensitivity do not account for such factors as distance to the tissue, differences in excitation light intensity, light collection efficiency, and differences in ambient light. The inevitable system variations that occur during quantitative comparisons of different tumor samples can be controlled for by using a ratiometric approach^33,34^. Herein, we have presented a method for quantitative fluorescence imaging that includes acquisition of the tumor PpIX fluorescence signal in the presence of a dye-in-polymer standard adjacent to the tumor, with subsequent image analysis using the open-source software FIJI (Carl Zeiss AG, Oberkochen, Germany). PpIX fluorescence intensity from the tumor and background was normalized to a selected dye-in-polymer standard (for example, standard 1 on Fig. 5). This method circumvents the effects of outside factors, other than field of view inhomogeneity. We applied this method for calculation of tumor fluorescence decay rates and compared it to the tumor-to-background ratios (Fig. 6).

The ratiometric method that uses a standard fluorescent material is simple and robust, in that it controls for outside factors that affect fluorescence and could be used in studies that aim to accurately measure fluorescence using operating microscopes. Dye-in-polymer standards can serve as a more reliable reference point to assess the relative intensity of the PpIX signal.

### Fluorescent Dye-in-Polymer Standards

The robust dye-in-polymer standards offer a portability that is convenient and reliable for comparing surgical microscope systems within an organization or among geographically separate institutions. These standards are made of uniform and stable materials, and they are fabricated in page-sized sheets. The dye-in-polymer technology allows them to be adapted to other targeting wavelengths by using appropriate dyes.

**Figure 7:**
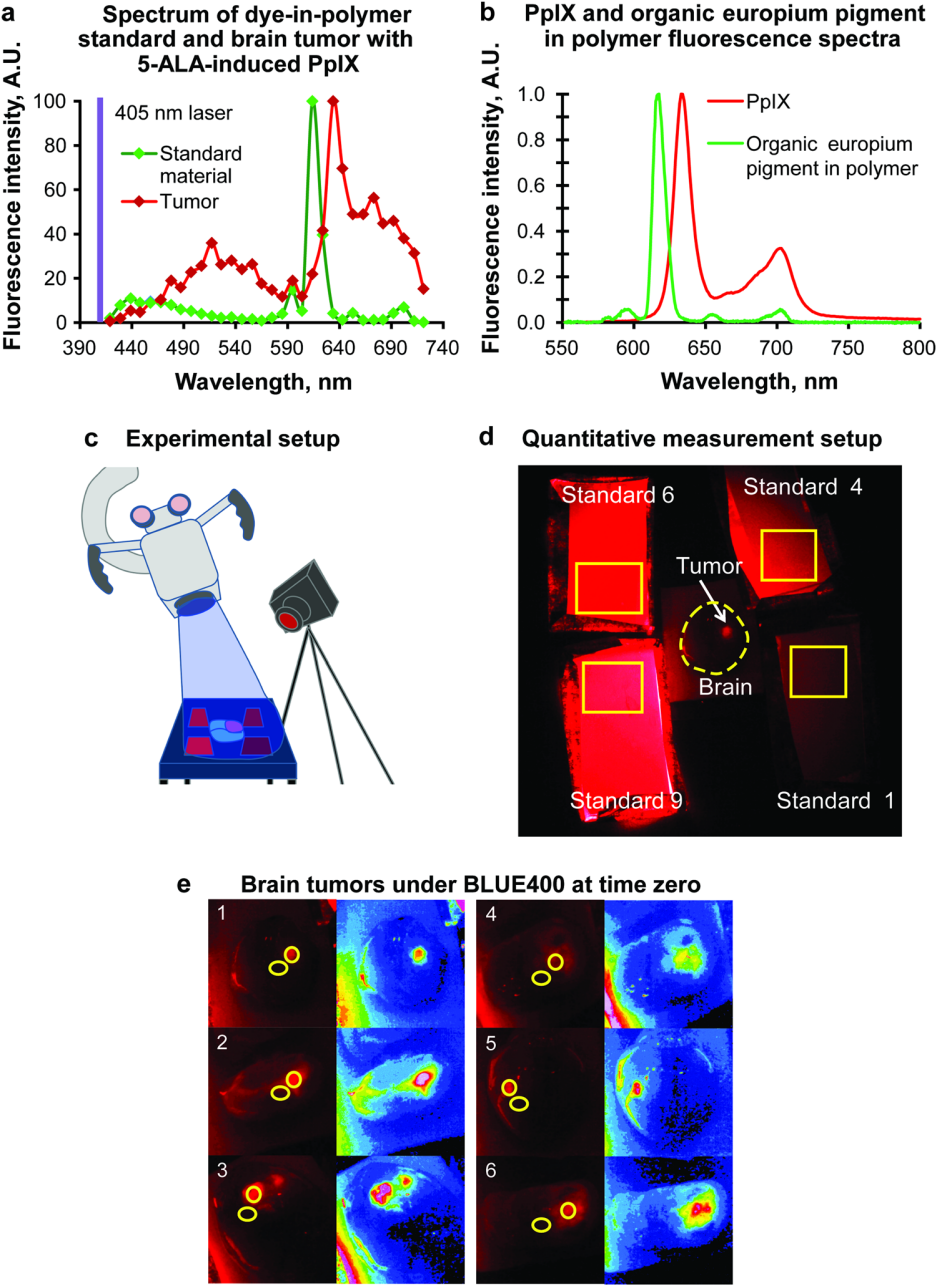
Experimental setup. (**a**) Fluorescence spectra of the dye-in-polymer standard and 5-ALA–induced PpIX in mouse glioma. All standards showed perfect alignment of the emission spectra. Results from standard 1 are presented on the graph. Measurements performed on a Zeiss LSM710 laser scanning spectral confocal microscope, EC Plan-Neofluar 10×/0.3 objective, and 405 laser using the lambda mode with 32-channel detector covering range 419–721 nm. Mouse glioma imaging was performed *ex vivo* immediately after brain harvesting, 2 hours after 5-ALA administration. Data are presented as normalized % values to the maximum peak value. (**b**) Fluorescence spectra of protoporphyrin IX (PpIX) dimethyl ester dissolved in chloroform and the organic europium pigment dispersed in polymer. PpIX data were downloaded from http://omlc.org/spectra/PhotochemCAD/html/149.html which used data from Photochem CAD package, version 2.1a (Du 1998, Dixon 2005). (**c**) Setup for quantitative measurement of the PpIX signal and photobleaching in glioma tissue. Operating microscope is shown at left, external camera at right. (**d**) Operating room setup for quantitative measurement of PpIX fluorescence and normalization to the standard. Yellow rectangles show regions of interest selected for analysis of standards. (**e**) Two regions of interest were selected on 6 mouse brains: 1 over the tumor and 1 over the normal brain. Left image shows raw red channel, right image shows corresponding channel in a heatmap lookup view. *A*.*U*., *arbitrary units; 5-ALA*, *5-aminolevulinic acid; PpIX*, *protoporphyrin IX*. *Used with permission from Barrow Neurological Institute*, *Phoenix*, *Arizona*.

It was important to fabricate standards with a range of fluorescence intensity to span the clinically observed extent of PpIX emission brightness. Hence, these standards not only were useful in benchmarking the animal model fluorescence intensity, but also were also useful in quantifying the variable human visual sensitivity to the range of PpIX fluorescence brightness. Furthermore, the sharp emission band of the organic europium pigment dispersed in the polymer standards closely matches the peak emission of PpIX fluorescence within ∼15 nm (Fig. 7a,b). All standards were very photostable, albeit with a slight change in fluorescence intensity after extended periods of blue light exposure (Supplemental Fig. 3). PpIX solutions could be prepared in advance to use as phantoms or standards^17,35^, but the solutions are not photostable and may require a separate sealing material to limit oxygen exposure. PpIX solutions that are exposed to air will photobleach^35^, which makes them less useful than the prefabricated dye-in-polymer sheets. Excellent stability and minimal photobleaching make the dye-in-polymer standard sheets a good reference material for quantitative imaging.

Another option for implementing a fluorescent standard consists of microspheres, which are routinely used in flow cytometry. In fact, europium-labeled microspheres are commercially available in a single concentration (Invitrogen FluoSpheres [F20881], Thermo Fisher Scientific; and FCEU, Bangs Laboratories, Inc.). However, the sphere diameters are about 500× smaller than the resolution capability of the operating microscope. Finally, the preparation of step-wise dye-loaded concentration batches is cost prohibitive. Although specialized technology exists to fabricate beads with a narrow distribution of fluorescent dye loading, the organic europium pigment dispersed in the polymer resin that was used in our study is inherently highly uniform.

### Rate of PpIX Fluorescence Photobleaching

Photobleaching of PpIX is usually not a concern during fluorescence-guided resection of high-grade gliomas. As resection progresses, new tissue layers with unbleached PpIX are exposed^36^. Furthermore, blood products and cottonoids can cover the area to protect it from photobleaching, although variations in surgical technique may not optimize the visualization of PpIX. However, awareness of PpIX photobleaching is an important concern during lengthy surgical resections (e.g., when the fluorescence intensity is weak at the beginning of the procedure or when the pace of tumor resection is considerably slowed or complicated). Decreased PpIX emission, especially at the tumor margins, may result in a false-negative interpretation. In these situations, the loss of fluorescence detection should be a concern when residual tumor at the margin has been bleached and there is no underlying tumor^36^. Since PpIX fluorescence can be obscured by ambient light, surgical resection is usually performed with the operating room lights dimmed. The detection sensitivity of the operating microscope can be increased by setting the xenon arc light source on 100% and positioning it at working distance of 25 cm. Other technologies that have sensitivity beyond the surgical microscope for detecting the PpIX emission include low-power scanning fiber endoscopes^26^, spectroscopy^14,37^, and quantitative fluorescence imaging^15^. It can therefore be assumed that a decrease in photobleaching would contribute to higher overall PpIX signal, which would facilitate detection^23^. Extension of imaging time without photobleaching of PpIX is possible with pulsed excitation^5^ and scanning technology^27,38^.

Stummer *et al*.^39^ assessed photobleaching of glioblastoma samples *ex vivo* with an early prototype of an operating microscope in violet-blue mode at a 25-cm working distance. They found that the fluorescence decay rate is variable: a decay to 36% (i.e., 1/e) occurs after exposure to 12.4 ± 8.6 J/cm^2^ irradiance, which corresponds to 25.7 ± 17.8 min (range 15.9–64.7 min) with the 8 mW/cm^2^ microscope light intensity^6^. Another study with a 17.8 mW/mm^2^ laser showed photobleaching of PpIX in *ex vivo* glioma tissue to 37% intensity within about 38 sec with continuous exposure and within 6 sec with pulsed laser irradiation^5^. We have also noted high variability in the rate of PpIX photobleaching in experimental gliomas that depended on the initial fluorescence intensity^26^. Such a large range of reported photobleaching decay is most likely due to glioma heterogeneity^40^. Another factor that affects PpIX fluorescence decay is the variability in light power output of the operating microscopes. We measured light intensity at a 25-cm working distance that ranged from 8.9 to 18.4 mW/cm^2^, which is higher than previously reported^6^. When the working distance is taken into account, the light power varied from a maximum of 25.2 mW/cm^2^ at 20 cm to a minimum of about 4.7 mW/cm^2^ at 35 cm in our observations (Supplemental Fig. 4). The resultant PpIX photobleaching rate per irradiance is in accordance with that reported in a previous study^6^; however, users should be aware of the variability of light intensity in current operating microscopes. We therefore recommend measuring the optical power of the fluorescent surgical microscope when performing quantitative PpIX studies, especially when different operating microscopes are used. Our study also demonstrated that the rate of photobleaching is uneven across the microscope field of view and that it depends upon the light power setting of the microscope.

### Operating Microscopes as a Source of Error in Quantitative Assessment of PpIX Fluorescence

Modern surgical microscopes have significantly advanced neurosurgery as a specialty and are currently in constant use in operating rooms worldwide. This study was not designed to question the efficacy of current methods of fluorescence-guided surgery using 5-ALA; however, it has exposed variations in the advanced functionality of, and the potential for, quantitative fluorescence detection with current commercially available clinical grade neurosurgical operating visualization equipment. Several aspects of current operating microscopes are worth discussing in relation to their effect on quantification attempts during fluorescence-guided surgery.

#### Excitation Light Source

First, the same xenon arc light source is used for both the white light mode and the blue light mode, which results in a faster depreciation of lamp output. Although we did not identify an association between lamp life time and detected light intensity, we cannot yet completely exclude this factor. The light output of xenon arc lamp illumination systems can prematurely degrade because of arc spot misalignment and because of evaporation of tungsten electrode material onto the light-transmitting lamp envelope; however, this was not investigated in this study. Second, the use of different microscopes and an assortment of remaining bulb life times may also introduce bias or alter the quantification of the PpIX fluorescence signal.

#### Illumination Surface Profile

Next, the operating microscope illumination beam pattern is not uniform, which leads to variations in fluorescence intensities and photobleaching rates across the field of view (Fig. 2). This aspect is particularly important and has not been addressed in current operating microscopes. Furthermore, a tumor resection cavity is not a flat and uniform surface, and those that are deep within the brain may require retraction for access and illumination, which exacerbates the biases inherent in comparisons of PpIX emission intensities.

#### Fluorescence Signal Detection Method

PpIX fluorescence observed through the oculars is reported to be superior to the images or videos recorded by the operating microscope^16,17,27,41^. This improvement is partly due to the beam splitter in the operating microscope design, which directs only a portion of the collected photons to the camera, and partly to the color separation limitations of the internal microscope camera itself. In this study, we used an external Canon CMOS camera equipped with an external red filter to fully reject blue light leakage. Newer versions of operating microscopes are expected to have more sensitive cameras for fluorescence detection. In addition to the need for higher resolution and improved sensitivity, improved color separation is necessary to avoid blue light leakage into the other imaging channels.

#### Distance

The intensity of the fluorescence is highly dependent on the focus distance, and it is highest for contact or near-contact microscopes or endoscopes^42–45^ because the light beam is cone-shaped and its intensity is a function of distance from the source. In previous studies, the distance from the microscope to the operative field was not well documented or noted, and it is unclear whether such analyses were performed with the same microscope each time or whether different microscopes were used^11,46,47^. These configuration parameters should be taken into account and documented in future studies.

#### Ambient Light

Ambient light in the operating room is another important factor to consider when conducting quantitative analysis. Ambient light can have a significant impact on the detection of fluorescence signal because it reduces or introduces extraneous red channel background light.

#### Techniques for Overcoming Limitations

Ultimately, the results of this study suggest that the recording of PpIX fluorescence for off-line analysis and quantification should be performed with a dedicated highly sensitive camera and also possibly with an LWP filter combination that provides a large dynamic range. In addition, fluorescence reference standards can further increase measurement accuracy and quantify interoperator relative sensitivity.

### Study Limitations

In this study, we measured the illumination properties of clinical grade operating microscopes using an optical power meter and fluorescence standards. We then applied these quantitative measures to evaluate a biological substrate (i.e., an experimental glioblastoma). The analysis of the tissue fluorescence led to a determination of the photobleaching rate of PpIX. We tested operating microscopes from only one commercial manufacturer, which may be an advantage because of their homogeneity. However, these methods may easily be applied to other microscope platforms with fluorescent functionality.

## Conclusion

In this study, we measured the spatial illumination intensity of commercial neurosurgical operating microscopes in standard white light and blue light modes. Illumination intensity was highly dependent on the distance to the sample tissue, the microscope light power settings, and the location within the field of view. Moreover, the various microscopes in our institution, which is a high-volume neurosurgery center, exhibited considerably different illumination optical power levels at identical system settings. Hours of remaining xenon arc lamp operation, based on a lifetime of 500 hours, showed no correlation to the variations in the measured optical power; however, the contribution of the remaining lifetime to the variability could not be completely eliminated. Altogether, these factors may impact the measured and visual appearance of fluorescence intensity in tumor and normal tissue. Qualitative and semiquantitative assessment of dye-in-polymer standards with spectral emission properties similar to PpIX demonstrated excellent photostability and good overall visual agreement among observers. Finally, we developed and described a ratiometric method for quantitative measurement of fluorescent signal from PpIX that accounts for various external conditions and variations in the different operating microscopes. Our findings about the limitations of quantification of PpIX fluorescence with neurosurgical operating microscopes potentially have considerable research and clinical implications.

## Methods

### Dye-in-Polymer Standard Red Fluorescent Material

Nine plastic transparent polymer sheets containing a dispersed proprietary organic europium pigment (Red Emitter 615; Oakley, Inc., West Chester, Ohio) of varying concentrations and thicknesses were fabricated for this study and are referred to as “standards” 1–9 throughout the manuscript (Table 4). The concentration is presented as parts-per-thousand (ppt) by weight. The standards were made from transparent liquid urethane resins and cell cast to A4 format (210 × 297-mm) sheets. A resin system was formulated to achieve a polymer glass transition temperature of 110 °C. Sheet samples of standards were provided by Korry Electronics Co. (Everett, Washington)^48^.

**Table 4:** Characteristics of standard red fluorescent material standards used in the study.

Dye-in-polymer Standard, No.	Thickness, Inches	Organic Europium Pigment Concentration, ppt by Weight
1	0.02	0.5
2	0.04	0.5
3	0.08	0.5
4	0.02	2.7
5	0.04	2.7
6	0.08	2.7
7	0.02	8.1
8	0.04	8.1
9	0.08	8.1

ppt = parts per thousand.

The fluorescence emission spectrum was consistent across the various concentrations and thicknesses of the standards when illuminated with a 405 laser. It exhibited peak fluorescence at 610–630 nm, slightly shorter than the PpIX emission wavelength in the tumor (Fig. 7a,b).

### Fluorescence Intensity Measurement of the Standards

The operating microscope (Pentero 900, Carl Zeiss AG, Oberkochen, Germany) in BLUE 400 mode was used a source of excitation blue light. The microscope was positioned at 20-cm focus distance (close to the minimally possible distance of 19.7 cm) and set at a 100% light power setting. Red fluorescence from the standard material sheets was recorded using the 18 megapixels Canon EOS Rebel T2i camera equipped with a complementary metal–oxide–semiconductor (CMOS) sensor and red long-wave-pass (LWP) filter (B + W F-Pro 58 090 5 × E from Schneider Kreuznach, Bad Kreuznach, Germany) positioned 75 mm away from the fluorescent imaging target. Camera settings (e.g., shutter speed, exposure, and focus) were optimized to reflect human eye experience and were kept unchanged. Images were also acquired using the internal color CCD camera of the operating microscope set at 15 frames per second to maximize sensitivity. Images were collected every 1 min for the first 10 min, and then every 5 min for the next 20 min.

### Microscope Optical Power Intensity Measurement Experiments

Nine Pentero 900 and one Kinevo 900 (Carl Zeiss AG, Oberkochen, Germany) clinical grade operating microscopes routinely used in the hospital operating rooms were assessed. Remaining bulb life time, focus distance, and light intensity settings were recorded from data displayed on the microscope work screen. Experiments were performed in darkened operating rooms. Microscopes were set up at constant distances from the target and incident light intensities were measured using a PM 200 power meter, and S120VC photodiode power sensor (ThorLabs GmbH, Dachau, Germany) calibrated for 405 nm. Optical power measurements were taken at various locations by moving the power meter probe across the round field of view of the microscope. A small aperture (6-mm diameter) was set in front of the power meter sensor to provide better granularity of the optical beam measurements. We recorded the optical power of the standard white light and the blue light in BLUE 400 mode. The zero point of the power meter was adjusted in each operating room before taking measurements to compensate for variations in ambient light.

### PpIX Bleaching Experiments in Glioma Model

Approval for the study was received from the Institutional Animal Care and Use Committee for St. Joseph’s Hospital and Medical Center and Barrow Neurological Institute, Phoenix, Arizona. The mice were kept in an institutional vivarium with a standard 12-hour day/night cycle and free access to food and water. The study was conducted in accordance with the *Guide for the Care and Use of Laboratory Animals* provided by the National Institutes of Health. All efforts were made to minimize animal pain, suffering, and reduce the number of animals sacrificed.

Experimental glioma models were established according to the previously described protocol in female 10-week-old B6(Cg)-Tyr^c-2J^/J mice, weighing an average of 20 g (The Jackson Laboratory, Bar Harbor, ME)^49,50^. Briefly, 2 µl of 1.45 × 10^7^ cells/ml GL261-Luc2 mouse glioma cells were stereotactically implanted in the right hemispheres after administration of a xylazine-ketamine anesthetic.

The surgeries and fluorescent imaging were performed in the surgical laboratory with the mice anesthetized (intraperitoneal xylazine 5 mg/kg and ketamine 50 mg/kg) with normothermia control, 30 days after tumor implantation and 2 hours after intraperitoneal injection of 5 mg of clinical-grade 5-ALA (Alasense, NIOPTIC, Moscow, Russia) dissolved in 200 µl of 1× phosphate-buffered saline. After the craniotomy, the brain tumor was exposed and the fluorescence standards were positioned around the brain. Continuous imaging with the operating microscope in BLUE 400 mode was performed within 30 min (Fig. 7c–e). At all time points, the microscope was set at a 20-cm distance and at a 100% blue light intensity.

### Image Analysis

Image analysis and quantification was performed in FIJI^51^ and ZEN 2.3 (blue edition) software (Carl Zeiss AG, Oberkochen, Germany). Images from both the Canon camera and the operating microscope camera were analyzed to generate objective evaluation of the fluorescence light intensity captured across the field of view.

To assess the photostability of the standards, we manually selected 10 different regions of interest (ROI) on each image. Each ROI was a circle with a 51-pixel radius. ROI locations were determined on the basis of data that reveal that the intensity of light is greatest within the center and reduced in the periphery in a nonuniform concentric pattern.

For the brain tumor PpIX photobleaching analysis, we selected six ROIs: four ROIs were placed over the standards 1, 4, 6, and 9, one ROI was placed over the tumor, and one ROI was placed at the brain adjacent to the tumor. Mean ± SD values of the ROI pixel intensities in the red channel were analyzed. The ratios of tumor to normal brain pixel values were also calculated. We did not subtract the small autofluorescence contribution to the PpIX fluorescence in our analysis.

### Qualitative Analysis of Fluorescence Intensity

Two neurosurgeons familiar with 5-ALA fluorescence-guided surgery and two trained medical students were asked to visually evaluate fluorescence intensity of the standards seen through the oculars of the operating microscope using a qualitative scale^52^ (Table 5). Standards were put under the BLUE 400 light, which was set at 100% light intensity and a 20-cm distance. The set of nine standards was evaluated by each observer and was presented twice as two random sequences.

**Table 5:** Scoring system for measuring visual red fluorescence intensity*.

Score	Definition
0	No fluorescence
1	Minimal fluorescence
2	Moderate fluorescence
3	High fluorescence
4	Very high fluorescence

*Adapted from Valdes *et al*.^47^.

### Statistical analysis

Interobserver and intraobserver agreement rates were calculated in Excel (Microsoft Corp., Redmond, WA) using the kappa statistic. Comparative analysis of fluorescence intensity was performed using the t-test. Data are presented as mean ± SD. P < 0.05 was selected as the threshold of significance.

### Data availability

Data are available upon request from the corresponding author.

## Electronic supplementary material

Supplementary figures
