# Supplementary material for: Optical Characterization of Neurosurgical Operating Microscopes: Quantitative Fluorescence and Assessment of PpIX Photobleaching
Source: Sci Rep. 2018 Aug 22;8:12543. doi: 10.1038/s41598-018-30247-6 (PMC6105612; doi:10.1038/s41598-018-30247-6)
Supplement: Supplementary file 1 — Supplementary figures [file 41598_2018_30247_MOESM1_ESM.docx]

**Optical Characterization of Neurosurgical Operating Microscopes:**

**Quantitative Fluorescence and Assessment of PpIX Photobleaching**

Evgenii Belykh, MD^1,2^

Eric J. Miller, BS^1^

Arpan A. Patel, BS^1^

Baran Bozkurt, MD^1^

Kaan Yağmurlu, MD^1^

Timothy R. Robinson, MS^4^

Peter Nakaji, MD^1^

Robert F. Spetzler, MD^1^

Michael T. Lawton, MD^1^

Leonard Y. Nelson, PhD^3^

Eric J. Seibel, PhD^3^

Mark C. Preul, MD^1*^

^1^Department of Neurosurgery

Barrow Neurological Institute

St. Joseph’s Hospital and Medical Center

Phoenix, Arizona

^2^Irkutsk State Medical University

Irkutsk, Russia

^3^University of Washington

Human Photonics Lab

Seattle, Washington

^4^RGB Optics

Lake Forest Park, WA

**Correspondence:** Mark C. Preul, MD

c/o Neuroscience Publications; Barrow Neurological Institute

St. Joseph’s Hospital and Medical Center

350 W. Thomas Rd.; Phoenix, AZ 85013

Tel: 602.406.3593; Fax: 602.406.4104

E-mail: [Neuropub@barrowneuro.org](mailto:Neuropub@barrowneuro.org)


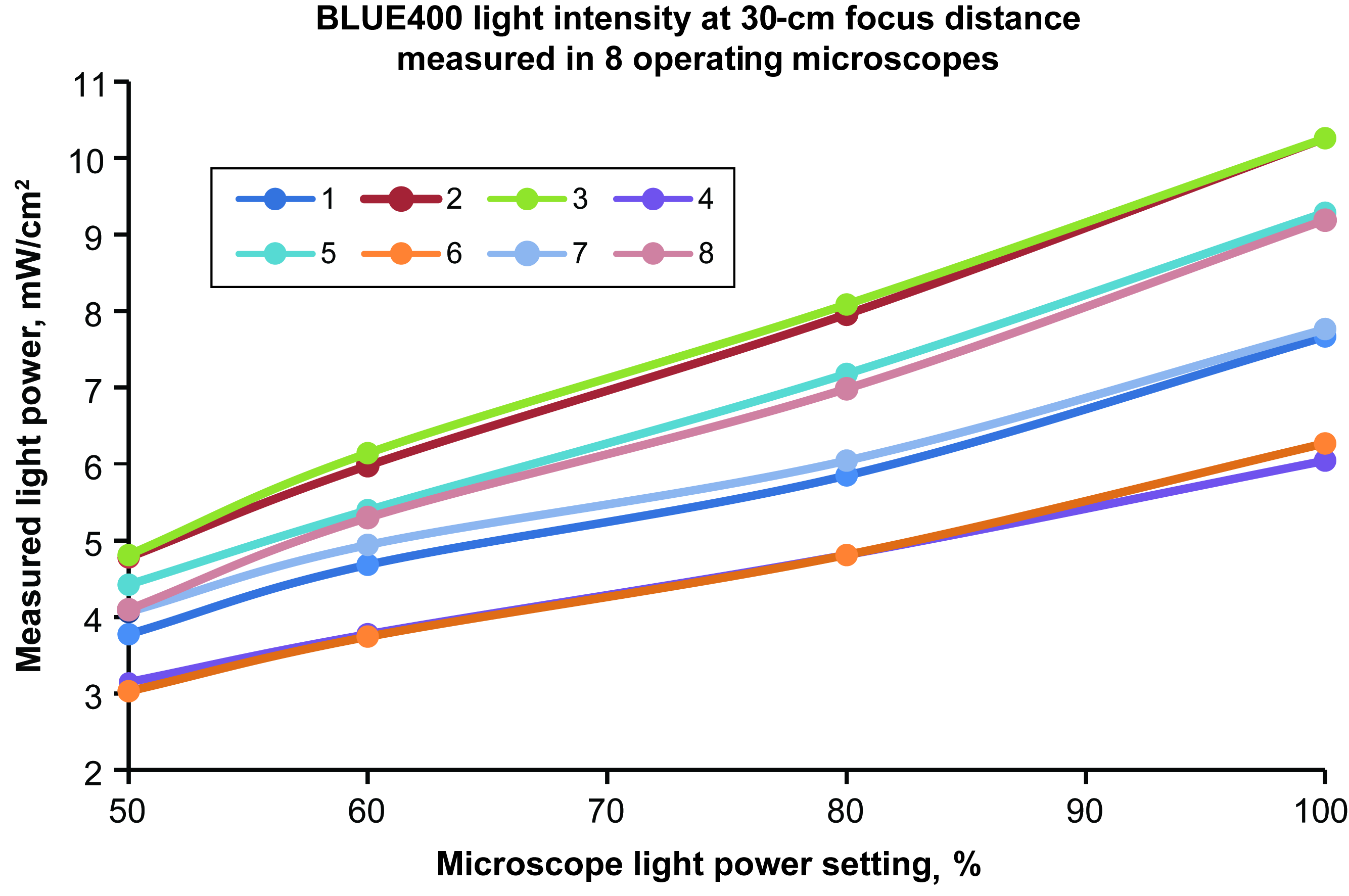


**Supplemental figure 1**. **Measured light power density in 8 operating microscopes used in BLUE400 mode.** The microscopes were positioned at 30-cm focus distance, and the light power setting was changed. *Used with permission from Barrow Neurological Institute, Phoenix, Arizona.*


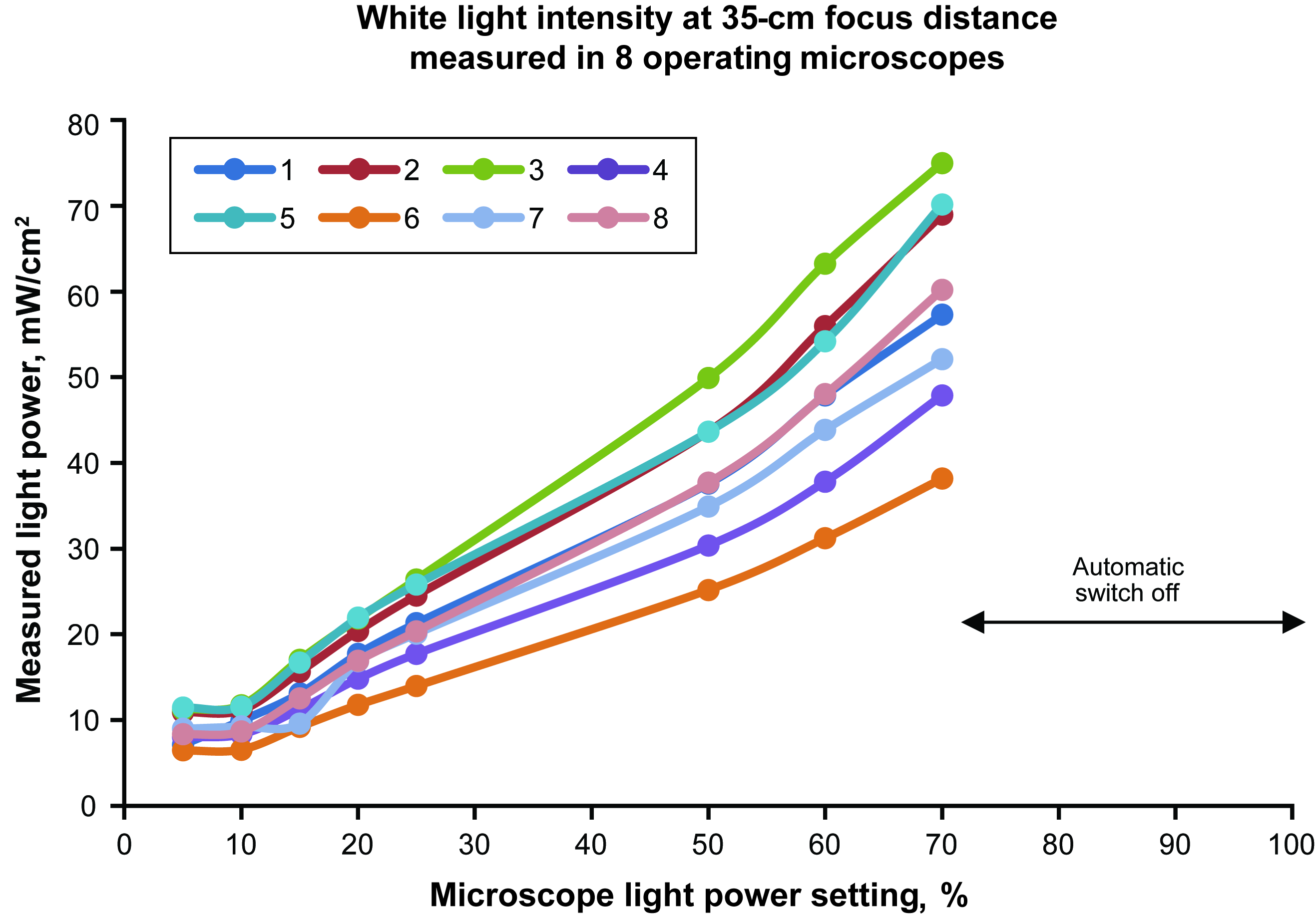


**Supplemental figure 2. Measured optical power density of the regular white light mode in 8 operating microscopes.** The microscopes were positioned at 35-cm focus distance, and the light power setting was changed. The microscope light switches off automatically when the intensity setting is increased more than 70%. Measurements revealed high variability among individual microscopes, with almost twice the difference in delivered optical power between the brightest and dimmest microscope. *Used with permission from Barrow Neurological Institute, Phoenix, Arizona.*


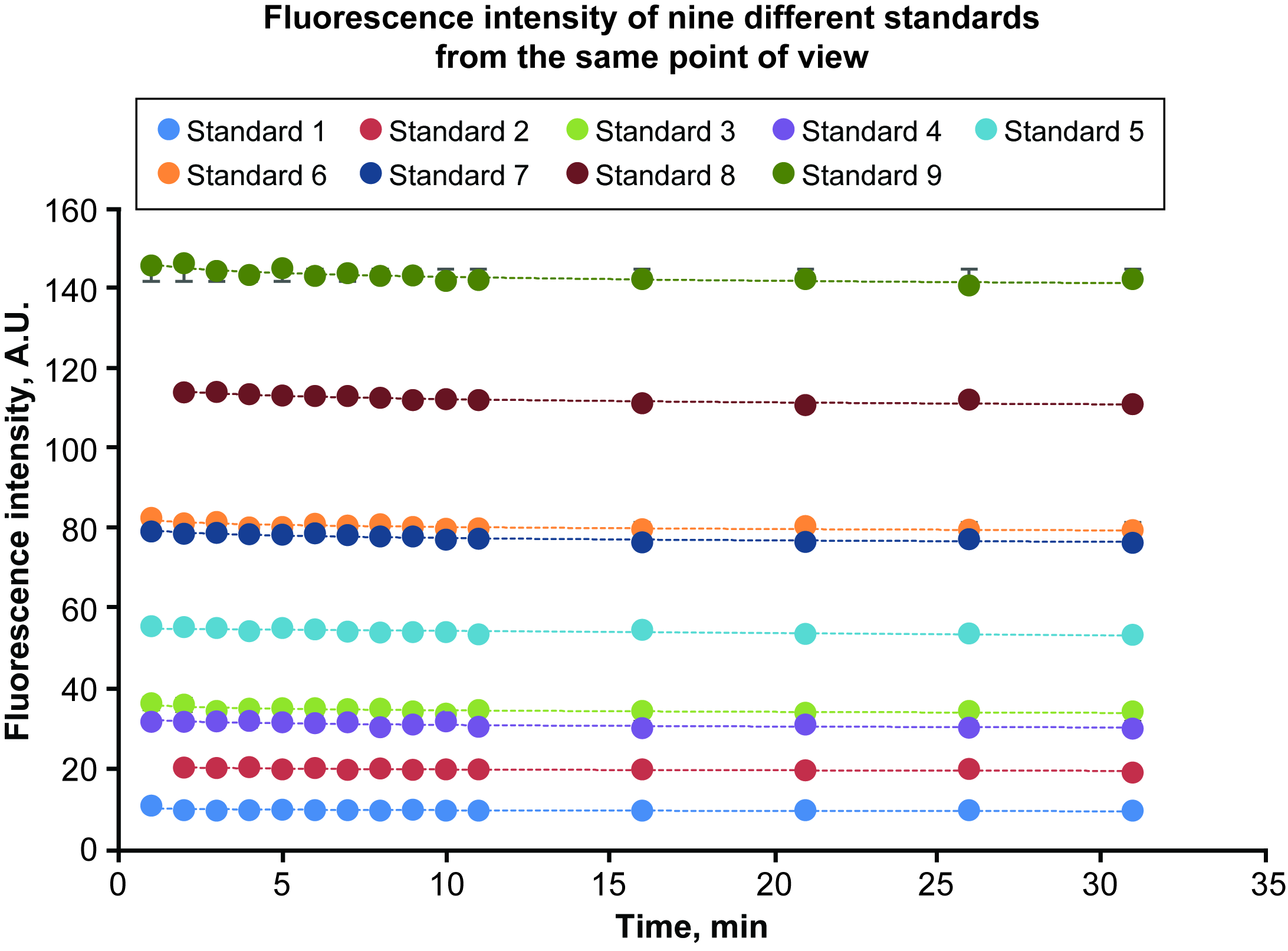


**Supplemental figure 3. Fluorescence decay rates for nine dye-in-polymer standards.** The intensity values are shown for location 1 (Figure 2D) for all nine standards over 30 min. *A.U., arbitrary units. Used with permission from Barrow Neurological Institute, Phoenix, Arizona.*


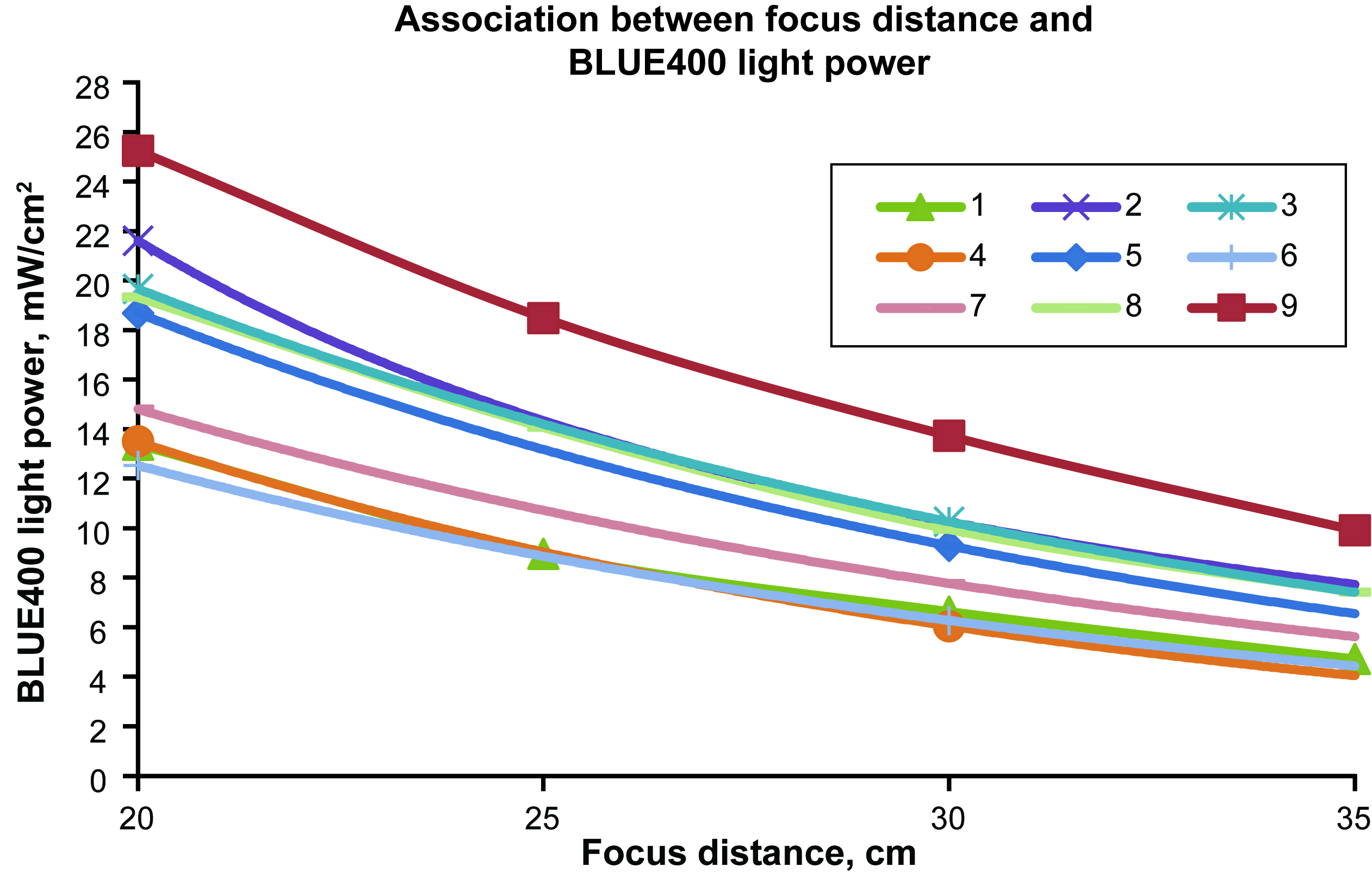


**Supplemental figure 4.** **Assessment of the relationships between the BLUE 400 optical power density and focus distance in nine operating microscopes.** *Used with permission from Barrow Neurological Institute, Phoenix, Arizona.*
